# How Midwestern College students protected their families in the first year of COVID-19

**DOI:** 10.3389/fpubh.2023.1143342

**Published:** 2023-06-02

**Authors:** Tyler W. Myroniuk, Michelle Teti, Ifeolu David, Enid Schatz

**Affiliations:** Department of Public Health, University of Missouri, Columbia, MO, United States

**Keywords:** college students, COVID-19, family, infectious diseases, public health, qualitative methods

## Abstract

**Introduction:**

College students routinely visit their families due to geographic proximity and their financial dependence. Consequently, the potential of transmitting COVID-19 from campus to their families’ homes is consequential. Family members are key sources of support for one another in nearly all matters but there is little research uncovering the mechanisms by which families have protected each other in the pandemic.

**Methods:**

Through an exploratory qualitative study, we examined the perspectives of a diverse, randomly sampled, group of students from a Midwestern University (pseudonym), in a college town, to identify COVID-19 prevention practices with their family members. We interviewed 33 students between the end of December 2020 and mid-April 2021 and conducted a thematic analysis through an iterative process.

**Results:**

Students navigated major differences in opinions and undertook significant actions in attempts to protect their family members from COVID-19 exposure. Students’ actions were rooted in the greater good of public health; prosocial behavior was on display.

**Discussion:**

Larger public health initiatives could target the broader population by involving students as messengers.

## Introduction

In the early stages of the COVID-19 pandemic, the international media showed American students partying on beaches and on campuses during Spring Break. Despite their awareness of, and exposure to, scientific expertise, students were not innate models of pandemic public health best practices (1–3). However, news outlets failed to capture college students’ on-and off-campus prosocial behavior—where they limited their own social activities and engaged in public health best practices to minimize COVID-19 transmission, for the benefit of society and their families (4). Because of students’ routine family visits, due to their geographic proximity to parents and their dependence on family members for financial and emotional support during their college years (5–7), the potential of transmitting COVID-19 from campus to their families’ homes is consequential. Nuance in college students’ COVID-19 risk mitigation strategies remains relatively unknown, as students likely faced numerous trade-offs in sustaining relationships and being mindful of public health.

Family members are key sources of support for one another in nearly all matters (8–11), including health decisions such as routine vaccinations (12–14), accessing antiretroviral treatment for HIV/AIDS (15, 16), and screening for degenerative diseases such as cancer (17, 18). Family members’ roles and support in the COVID-19 pandemic—while potentially like the circumstances surrounding other health issues—remains comparatively less known since it is a novel disease with little extant research in this area (19). Initially, healthcare workers faced an ethical dilemma of contemplating refusing to work to protect their family members from contracting COVID-19 (20). Family members also faced a moral dilemma to not visit or care for relatives in nursing home and older adult care facilities (21–23). Notably, fostering positive relationships with older family members has helped college students cope with pandemic-related stresses (24). But beyond this, there is little research uncovering the mechanisms by which families have protected each other in the pandemic.

We examined the perspectives of a diverse group of students from a Midwestern University (MWU—a pseudonym), in a small town, to identify COVID-19 prevention practices with their family members. Our semi-structured interview data were collected at a crucial juncture between the end of December 2020 and mid-April 2021, with a widely available vaccine on the horizon and a year’s worth of students’ experiences managing the COVID-19 pandemic with their families. During the time of our study, this Midwestern town, in conjunction with MWU, enacted standard COVID-19 restrictions, such as wearing masks in public spaces and social distancing, based on scientific guidance from national and local public health leaders and stakeholders. The state, however, never mandated wearing masks, social distancing, or other commonsense protocols to prevent COVID-19 transmission; after this study ended, the state took extraordinary legal efforts to prevent local level jurisdictions to mandate any COVID-19 prevention mechanisms. Students like those at MWU are important in quelling the spread of COVID-19 due to their transience between their college town and “home” residences (25). No students interviewed considered this small town their home because their parents—who they tended to visit during holidays or special events and live with outside of the academic calendar year—lived in other parts of the state or beyond. These “home” residences were usually at least an hour away—as is common in small American “college towns.” Given students’ exposure to strict campus COVID-19 protocols and constant COVID-19 prevention messaging from university leaders, we ask here: did students engage in best prevention practices away from campus with their family members? Students navigated major differences in opinions and undertook significant actions in attempts to protect their family members from COVID-19 exposure, with varying degrees of success. The objective of our study is to provide the scientific community much needed, in-depth, insight into the conversations about COVID-19 prevention and the extent to which strong relationships were tested during a time of great uncertainty.

## Materials and methods

### Study design

Our exploratory qualitative study data come from Midwestern University (MWU) students who initially took part in the *MWU Study of Seropositivity and Risk for SARS-CoV-2 and COVID-19* (ethics approved under University of Missouri, IRB protocol 2028427). We interviewed 33 participants, drawn from 1,155 students who took part in a survey, agreed to be part of follow-up studies, and provided an email address to be contacted at regarding follow-up studies (the total number of survey participants, which also included faculty and staff was 2,894). Student participants were not selected based on any particular degree program. Through a “nested” sampling design (26) stemming from the representative MWU student data—an advantage over conventional convenience or snowball samples—we randomly selected students (including both undergraduate and graduate) to interview from three different groups to ensure diversity of the sample: (1) an initial 10 students regardless of individual characteristics (sampled from *N* = 1,155); (2) 13 non-White students (sampled from *N* = 117); and (3) 10 LGBTQ+ students (based on self-identification; sampled from *N* = 151). Gender, ethnicity, and sexuality are relevant to the American student context and key to this stratified random sample.

Our sampling strategy aimed to maximize the heterogeneity of our sample to reflect the experiences of the diverse student body and achieve saturation among the ideas of different demographic groups. Also, based on our prior experience curating qualitative samples, we believed that the number of participants in each group would give us a high probability of reaching saturation in COVID-19 experiences and perspectives (27)—which ended up being the case with our data. Of the 33 students interviewed, 20 identified as female (60.6%), 12 as male (36.4%), and 1 as non-binary (3.0%). Along racial lines, 17 participants identified as non-White (51.5%), while 16 identified as White only (48.5%). Further, 23 participants identified as heterosexual (69.7%), with the other 10 participants identified with another sexuality (30.3%).

### Analytic techniques

We conducted individual semi-structured interviews, using a script, over Zoom with the 33 students which lasted between 35 min and 1 h; three of the authors conducted all interviews. The interviews were recorded and transcribed. Students were asked broad questions about the COVID-19 pandemic, including how it had generally changed their lives, whether they knew anyone who was infected with COVID-19, their worries about their futures and family members, what they do to protect themselves from becoming infected, the lessons they learned about themselves in the pandemic, and what colleges need to know to better understand student needs, among others. Using qualitative methods, we conducted a thematic analysis (28–31) through an iterative process with the aim of assessing how students navigated the COVID-19 pandemic and managed relationships with family members. All authors read the transcripts and independently generated a list of initial general themes in the data around COVID-19 experiences and prevention. Next, the team met to compare and discuss their lists. In this process we expanded, consolidated, and redefined our initial ideas, and then created a more-targeted master list of themes that we defined in a codebook for further exploration in the data—including the family-oriented themes described in the results section. We engaged in multiple test coding and debriefing sessions to resolve discrepancies to ensure trustworthiness of coding between authors (32). Authors then coded the transcripts via the codebook using Atlas.ti.

We presented our results with a participant pseudonym, gender identity (M, F, NB), White/non-White racial/ethnic indicator (W, NW), and month of interview. However, in this paper, we did not break down our themes along these characteristics. While our analyses did not uncover meaningful differences across these categories, participant characteristics provided context and conveyed similarities experienced by all students despite gender and racial differences of our respondents.

## Results

This paper focuses on two primary themes that emerged from the data during our co-analyzed qualitative thematic analysis method: (1) Differing Perspectives and (2) Protective Actions. Within these major themes, we also identified several prominent sub-themes through this method—found below. To contextualize our findings, the MWU student body was certainly at risk of transmitting COVID-19 during the time of this study—the end of December 2020 to mid-April 2021—because of limited existing or enforceable COVID-19 prevention policies (outside of clinical settings) due to state-level politics; COVID-19 prevention efforts were consequently limited on the MWU campus. Most students in our sample were employed at least part-time, mainly in service sector positions too (such as at restaurants, department stores, or grocery stores), with only a few having worked in a hospital setting at any point since the onset of the pandemic. Nearly all students lived away from their parents and grandparents during the academic year—when this study took place. Generally, the results indicate that students undertook best practices to prevent COVID-19 transmission but doing so took an emotional toll on them.

### Differing perspectives

The sheer difficulties of maintaining family life amid the global pandemic led to contention over views of how to sustain relationships. Some students expressed concern over how their family members were taking less COVID-19 precautions as them—due to political, regional, or otherwise inexplicable reasons. Other students described their family members going above and beyond best preventative practices which raised students’ anxieties about the pandemic.

#### Politics

The constantly changing, polarizing, American political debates about the severity of COVID-19, mask mandates, and social distancing contributed to intra-family differences in protective practices. Family members who participants identified as politically conservative—which was substantial in the conservative-leaning state that our research was based in—were most at odds with students:

It is a point of contention between my stepdad and I. My stepdad is very conservative and is on the like, ‘we don’t need to wear masks,’ kind of a train. I don’t know how my mom deals with it … but I think he’s starting to come around (Ana, Female, NW, Feb2021).

While Ana was slightly optimistic about her father, other students, like Jasmine and Rebecca, were not and depicted serious arguments with their parents. Political-based divisions within families, over COVID-19 prevention, were not trivial; irreconcilable differences in opinion between students and parents led to painful conversations:

My parents live ten hours away … in Texas. And Texas, especially right now, is not the best place for the pandemic, and throughout this whole thing has been a fiasco. I feel like our relationship has been kind of strained a little bit. I went home a few weeks ago for my brother’s senior night and ended up yelling at them for 45 minutes about not following COVID practices properly. And so, that coupled with last summer, when everything was happening with Black Lives Matter as well as the pandemic—a lot of tension there … just because they have a ‘difference of opinion,’ as they put it, on a lot of the science, which is kind of annoying (Jasmine, F, W, Apr2021).

On a few occasions, these intra-family divisions spilled out in public spaces. In Rebecca’s case, she could no longer hold back her true feelings about her dad’s COVID-19 choices:

I worry about my dad, who’s a paramedic, who’s constantly exposed to it. Doesn’t always take proper precautions when he’s not working, which I find very interesting … I’m from [a small town], which is like 20 miles west of here … A lot of people that think all this is a hoax. And my dad does lean conservative … He went to Walmart without a mask, and I was trying so hard to not yell at him, but I yelled at him. I was like, ‘What are you doing? I’d rather you not die. Thank you’ (Rebecca, F, W, Jan2021).

#### Cognitive dissonance

Not all family members’ and students’ discordant views were based on politics; these other differences were inexplicable, which perplexed and frustrated students. Lauryn (F, NW, Jan2021) could not reconcile her mom’s educational attainment and belief in misinformation as she described, “A lot of it I hear from my mom, and she is a scientist, but she is also on WeChat a lot … a lot of misinformation gets spread around. We are like ‘mom you are a researcher; you should know this!’” This cognitive dissonance was surprising to Lauryn because of the perception that scientific training and COVID-19 best practices adherence were congruent with one another. Grace (F, W, Jan2021) witnessed this cognitive dissonance in her sister, who also attended MWU. They were both exposed to the same campus and community COVID-19 prevention messaging, but Grace’s sister generally disregarded safety measures for no apparent reason:

You know, she was seeing small groups of people, but those people were seeing other people, so the circle got larger … I stopped hanging out with her just because I wanted to be safe and Thanksgiving break was coming up. I had a conversation with her. I was like, ‘You know, you’re going home to mom and dad. They’re at risk, you know?’ She was like, ‘Oh, I’m being fine. You know, whatever.’ And then she comes home for Thanksgiving, and she tests positive for COVID …

It remains unclear whether the intra-family feuds arising from not following public health guidelines persisted, but respondents’ passionate descriptions of these exchanges suggest fundamental differences that continue to divide Americans as the pandemic progresses.

#### Misunderstood messaging

There were enough instances of frustrated students whose parents were perceived to go too far above and beyond best public health practices, to warrant a theme. However, these differences were not based in politics but disagreements over how best to prevent COVID-19 transmission.

When at home with his mom, Alex (M, NW, Mar2021) described her as “going crazy and we’d disinfect all her groceries before coming in and stuff. But like, once I came back to MWU, I did not do all that.” Grace (F, W, Jan2021) also described her dad—who has some health issues—as “crazy” because “he does not leave the house … Whenever we are around him, he’s like, ‘Stay away from me.’”

In another example, Alexis (F, W, Jan2021) understood her family’s source of reasoning for being so cautious in the pandemic, but implied she was not as strict as them:

My family like I said—my dad being a doctor—since the beginning of the pandemic we have taken it really seriously. I think my mom was wanting to wear a mask before it was the thing to do. People would look at her weird in the store because no one thought that was what you were supposed to do at the beginning of the pandemic, but we did that early because of my dad’s knowledge. My family has always taken it seriously. My dad’s parents have only done curbside getting food or groceries. They have really sheltered in.

Theo’s (M, NW, Feb2021) mom went as far to put an end to his pursuit of employment by saying “no, you cannot get a job. You’re going to get COVID and you are going to bring it here or you are going to give it to all of us.”

Students who were in these situations may have been annoyed at their family members, but these exchanges conveyed that students’ and their families’ influences on one another were complicated and multi-directional.

### Protective actions

Students discussed their prosocial behavior and attitudes, i.e., the desire to protect others, taking two primary forms: limiting visits or conducting visits at a distance or virtually. Even though many had relatives who worked in health care or other high-exposure jobs, students perceived their potential exposure at college as high and did not want to be the one to bring COVID-19 “home.” To ensure that their loved ones—and themselves—stayed safe and healthy, they only reconsidered these behaviors once a vaccine was available.

#### Limiting exposure

Nearly all our respondents took proactive steps to reduce the risk transmitting COVID-19 to their families by limiting how many times they physically visited throughout the year. Jeff (M, W, Mar2021) took a hardline stance and would not even see his grandparents when visiting home. He made this decision out of an abundance of caution “because me being a college student, I’m exposed way more. Me at work, I could be exposed and not know it.” He saw his risk and potential for exposure as greater than that of his grandmother. This delay in seeing loved ones so as not to infect them sometimes came with a heavy price. Grace also did not visit her grandparents, as she explains, “Oh, I was saying both my grandmas, I have not seen them in a year. One of my grandmas actually passed away and that was difficult because I had not seen her in a long time.” (Grace, F, W, Jan2021).

Even nearby siblings were avoided by some due to common exposure risks. Lucas (M, NW, Mar2021) justified his distance from his family by saying, “I just do not know where they have been. They do not know exactly where I’ve been. We’ve been at school with all these other university students. So, neither one of us can say without a doubt that we are completely clean.”

Social distancing and virtual visits were not necessarily easier, as students implied some sadness and concern with this approach. Jordyn’s (F, W, Mar2021) somber tone when describing Christmas was emblematic of the feelings of many students. She explained, “we sat outside with masks and exchanged gifts with my grandparents. That was all we did. I have not really seen them in a while.”

Over the semester in which the interviews were conducted, the COVID-19 vaccine became increasingly available to older and immunocompromised individuals and then to the general public. Over this period, we identified a change in optimism regarding visits with older family members and potential future visits. Madison (F, W, Apr2021) summed this feeling when she stated:

So, my family was really worried about my sister getting it, because she has viral-induced asthma that’s pretty bad. But she was able to get the vaccine. She got a referral from the doctor to go get it. That was a big thing. My grandparents, obviously, but they both had the vaccine. You know, so, they’re a little bit less worried now, but definitely before they got the vaccine, they were all very concerned about getting it and passing it to other people.

Madison and others felt that their choices opened a bit more and their guard relaxed when those they worried about had access to vaccines, and thus had more protection if they were to get COVID-19.

Still, taking significant actions to protect family members at times came at a cost to relationships—some more outwardly evident than other, particularly when the person did not understand the motivation for staying separate. Rebecca (F, W, Jan2021) noted that the pandemic “has put a strain on some of my relationships, particularly with one of my family members who was very upset that she could not see us for Christmas. It is like, ‘I know that you have not been taking this seriously, and I love you, and I’m happy to schedule a Zoom call with you, but I’m not going to see you in person.’” As we show in the next section, differing levels of “taking [COVID-19] seriously” impacted the ways that students felt about relationships with their families.

#### Twinges of conscience

MWU student participants made it clear that they embraced protecting their immediate and extended family members from COVID-19, especially those in ill health. Underscoring all these were students’ worries, fears, and guilt of potentially transmitting COVID-19 to relatives given the high risk of exposure that students faced on campus. Given the news in the early days of the pandemic that older persons were especially at risk, students were particularly concerned about infecting their grandparents. Madison (F, W, Apr 2021) described her worries about getting COVID-19, not just because of her own health, but because of concern for her grandparents, “I was worried about others getting it … My grandpa has heart issues … my grandma broke her hip over the winter.” Similarly, Sebastian (M, NW, Mar2021) was concerned for his grandparents and parents—who live together—because in mid-March 2021 “none of them ha[d] gotten the vaccine yet,” so he worried about the consequences of them getting sick.

For others like Isla (F, NW, Feb2021), the stakes were, arguably, even higher:

My dad has one kidney, asthma, and he works in the healthcare field. My mom also works in the healthcare field. My stepdad has a horrible heart. My sister has an autoimmune disease. We have a lot of things going on in the family. During the summer I was very worried about giving COVID to my family. During the first semester I wanted to go back home, and it was difficult for my parents to tell me no that they couldn’t have me home until winter break … it is hard to not go home when I want to and hard to be so cautious.

While Isla thought it was important to protect her at risk family members, it was challenging for her that being cautious meant not being able to see them.

Students prioritized their family members’ health concerns over their own. Shawn (NB, NW, Apr2021) indicated where his concerns lay when saying “I’m not even that concerned with my health. I’m just concerned with passing it to my mom, dad, and my niece. Because even though they have taken precautions too, I interact with them the most when I go back home.”

Students’ actions were often spurred explicitly by the imagined guilt that would be felt if they infected their families. This duty and potential guilt were enough to keep students away from family members, even those who live alone and wanted the social connection of visiting family; Courteney (F, W, Jan 2021) asked, “What if I have it and I do not know, and I end up giving it to her, and things like that?”

Alexis (F, W, Jan2021) shared how stories her father told her made her worry about being the one to infect her family members. She said,

My dad is a doctor … he saw some patients die because their children brought COVID to them and so I was kind of worried about that—seeing my grandparents and being the one that would bring COVID to them. You know? That has been my biggest concern over time.

Ashley (F, W, Apr2021) conveyed an even heavier form of guilt, due to the size of the town her mom resides in she “would be worried about getting COVID, going home, passing it to my hometown, because we are not a very big town. Like, if my mom got it, the rest of our community would, too.” This sense of being bringing COVID-19 home weighed heavily on students during the 2020–2021 academic year, making them make different choices than they would have otherwise.

## Discussion

At the time of data collection, our study was (and continues to be) novel because it emphasized the role of students within their extended families in navigating the COVID-19 pandemic. Most studies of college students’ health have focused on depression, stress, and limited physical wellness routines amid massive career uncertainty (33–39). Although such studies are undoubtedly important, the exposure to evidence-based policies and discussions of COVID-19 at institutions of higher education gave students a unique perspective on the pandemic; this infection prevention information was transmitted to extended family members during a time of disinformation and health vulnerability. Thus, the key role of college students within their extended families ought to be better understood. Therefore, our work is an important contribution to this field.

Our findings suggest that the students who participated in our research understood the gravity of the pandemic, even in its early stages. For the most part, students were engaged in a myriad of COVID-19 risk mitigation strategies that they thought about and acted upon in relation to their families. Some students seriously worried about family members not taking the virus seriously enough, and for some this even led to strained relationships. Sometimes when comparing their own strategies with their families they saw their families as over-zealous, but few thought risk mitigation was unnecessary. To manage their own worries about family members, students took practiced social distancing and limited in-person visits, especially with respect to their grandparents. Students’ actions were not only self-serving but also rooted in the greater good of public health; prosocial behavior was on display.

Our research supports and builds on the limited existing research on how families protected each other from COVID-19 infection early in the pandemic. Overall, it was not surprising that students genuinely cared for their family members’ well-being and took actions to prevent COVID-19 transmission. However, the extent to which students pleaded and negotiated with parents to improve their prevention methods—rather than simply estranging themselves from their parents—implies that “blood” is indeed “thicker than water;” supportive family ties in times of health crises have rarely been as stringently tested in prior work with less contentious (though no less serious) prevention/treatment discussions around vaccinations and diseases (12–14, 17, 18). With their grandparents, though, students faced the moral dilemma that family members were documented to have faced early on in the pandemic—balancing the benefits of visiting and interacting with potentially frail individuals to show support and boost morale, or keeping away and not risking transmitting COVID-19 to the older family members (21–23). The student participants took strong precautions in avoiding contact with their grandparents but implied regret and sadness, as found elsewhere (24), in doing so because these self-imposed restrictions came at the expense of maintaining or building upon such important relationships. The detailed recollection of COVID-19 conversations provided by participants offers in-depth, previously unidentified, insight into the ways in which families grappled with the major, life-altering, shock of a global pandemic.

Based on our analyses, it appears that the parents, not grandparents, of students, mostly from Generation X—those born after the Baby Boomers but before Millennials—were the primary source of COVID-19 contention. The parents of young adults are a sizeable and possibly key demographic whose attitudes and behaviors could be changed regarding public health best practices; we found no discussions of contentious discussions where students’ strongly encouraged grandparents to engage in best prevention practices, compared to parents, implying that grandparents espoused similar views on pandemic health risks as students did. We can only speculate about the mechanisms leading to these results, but the oldest generations of Americans experienced major public health crises, including the polio epidemic, which could have influenced their behaviors. These findings push the existing literature which found that Millennials and those in Generation Z were more receptive to COVID-19 information (and misinformation) online, even if Generation X and older were more receptive to COVID-19 vaccination in general population studies (40–42). When considering MWU’s location in the Midwest, in a state with little political appetite to impose stringent public health policies, college students could be a practical means for spreading information and adopting practices that could mitigate the spread of COVID-19 and other viruses. Systemic, multi-institution research is needed to see if these results are robust. Larger public health initiatives could then target the broader population by involving students as messengers.

Our study has important limitations. First, it is unknown whether this prosocial behavior reflects all students’ perspectives at MWU even though we acquired a diverse sample of students from a larger, stratified randomly sampled student population. Of course, variation across universities within and between states is unknown too. Second, there is likely selection bias among those who participated in this qualitative study—by indicating their interest in doing so from the prior *MWU Study of Seropositivity and Risk for SARS-CoV-2 and COVID-19* survey—compared to those who opted-out or never participated in the prior study to begin with. Participation could be a proxy for willingness to acknowledge and discuss the realities of the COVID-19 pandemic—leading to a sample of the most proactive individuals and masking broader differences across demographic characteristics. Third, we cannot identify if social desirability influenced participants’ responses. Participants may have altered their responses regarding COVID-19 preventative actions to present themselves as engaging in best practices and doing the most they could to protect family members, with the purpose of presenting oneself to the interviewer as someone practicing socially acceptable pandemic behavior. Nonetheless, these data offer an important vantage point into the range of students’ mindsets in the throngs of the pandemic—students were generally responsible public health stewards, not liabilities who focused on partying above all else, as was depicted early on.

Students in our study generally engaged in best COVID-19 preventative practices and our detailed data convey the important role that students played in their extended families’ health decisions during the pandemic. We hope, in the interests of public health, that our students’ perspectives on preventing COVID-19 infections are, in fact, widespread and representative. This would bode well for future American public health initiatives around subsequent COVID-19 variants, influenza season, and future pandemics given the prevalence of young Americans who attend college and their dual relationship with the towns they temporarily reside in and their permanent homes where their families live.

## Data availability statement

The original contributions presented in the study are included in the article/supplementary material, further inquiries can be directed to the TM (tyler.myroniuk@health.missouri.edu).

## Ethics statement

The studies involving human participants were reviewed and approved by the University of Missouri Institutional Review Board Protocol #2028427. The patients/participants provided their written informed consent to participate in this study.

## Author contributions

TM, MT, and ID: conceptualization, data curation, formal analysis, investigation, methodology, software, writing—original draft, writing—review and editing. ES: conceptualization, data curation, formal analysis, investigation, methodology, software, writing—original draft, writing—review and editing and funding acquisition. All authors contributed to the article and approved the submitted version.

## Funding

This work was supported by the University of Missouri CARES and from SIEMENS Healthineers.

## Conflict of interest

The authors declare that the research was conducted in the absence of any commercial or financial relationships that could be construed as a potential conflict of interest.

## Publisher’s note

All claims expressed in this article are solely those of the authors and do not necessarily represent those of their affiliated organizations, or those of the publisher, the editors and the reviewers. Any product that may be evaluated in this article, or claim that may be made by its manufacturer, is not guaranteed or endorsed by the publisher.
